# Effects of the level of household access to water, sanitation and hygiene on the nutritional status of children under five, Benin

**DOI:** 10.1186/s40795-023-00751-8

**Published:** 2023-08-01

**Authors:** Nicolas Gaffan, Alphonse Kpozehouen, Cyriaque Degbey, Yolaine Glele Ahanhanzo, Moussiliou Noël Paraïso

**Affiliations:** 1 Department of Epidemiology and Biostatistics, Regional Institute of Public Health, University of Abomey-Calavi, Ouidah, Benin; 2 Department of Environmental Health, Regional Institute of Public Health, University of Abomey-Calavi, Ouidah, Benin; 3 University Hospital Hygiene Clinic, National Hospital and University Centre Hubert Koutoukou Maga, Cotonou, Benin; 4 Department of Health Promotion, Regional Institute of Public Health, University of Abomey-Calavi, Ouidah, Benin

**Keywords:** Child, DHS, Water, Sanitation, Hygiene, WASH, Stunting, Wasting, Underweight, Benin

## Abstract

**Background:**

Whether or not the Water, Sanitation and Hygiene (WASH) conditions in which children under five live determine their nutritional status is still under discussion. The work aimed to study the effects of household WASH conditions to which children under five are exposed on their nutritional status in Benin.

**Methods:**

The study utilized a cross-sectional design and consisted of secondary analyses using datasets from the fifth Demographic and Health Survey (DHS-V) conducted in Benin. Stunting, wasting and underweight were the dependent variables. The WASH conditions in which children live were evaluated in the immediate environment, i.e., at the level of their households. After describing the study variables, the relationships between the dependent variables and the exposures were checked using multivariate logistic regression. Data analysis was performed with Stata 15 and took into account the survey’s sampling design.

**Results:**

The prevalence of stunting, wasting and underweight was 31.15% (95% CI = 29.90-32.42), 4.79% (95% CI = 4.33–5.31) and 15.82% (95% CI = 14.92–16.76), respectively. The stunting odds were 1.35 (95% CI = 1.15–1.59) and 1.27 (95% CI = 1.01–1.59) times higher for children from households with no water and sanitation services, respectively, compared to children living in households with basic water and sanitation services. Children under five from households with no hygiene facilities and using limited hygiene services had 1.31 (95% CI = 1.05–1.63) and 1.35 (95% CI = 1.10–1.67) times the odds of being stunted, respectively, compared to children covered by basic hygiene facilities. There is no evidence of a significant relationship between household access to WASH and wasting in children under five. The odds of being underweight were 1.33 (95% CI = 1.02–1.72) times higher among children under five from households with limited hygiene facilities than among children from households with basic hygiene facilities.

**Conclusion:**

Interventions to fight malnutrition in children under five should include a WASH dimension.

**Supplementary Information:**

The online version contains supplementary material available at 10.1186/s40795-023-00751-8.

## Background

Malnutrition refers to an imbalance (deficiency or excess) in an individual’s energy or nutrient intake relative to normal physiological requirements [1]. While adults generally suffer from excess malnutrition (overweight or obesity), children, notably those under five, are more affected by undernutrition in its various forms, including stunting, wasting and underweight [2]. Globally, 149, 45 and 85 million children under five (CU5) are stunted, wasted and underweight, respectively [2, 3]. Although all regions are affected, Africa and Asia remain the continents with the highest cases. Indeed, more than half of all stunted CU5 live in Asia and two out of five in Africa [4]. Also, more than two-thirds of CU5 with wasting live in Asia and more than a quarter in Africa [4]. Finally, almost half of underweight children live in Asia and nearly a third in Africa [3]. According to a meta-analysis in 2017, the prevalence of stunting, wasting and underweight were 33.20%, 7.10% and 16.30% among CU5 in Sub-Saharan Africa, respectively [5]. In Benin, according to data from a national survey, 32% of CU5 were stunted, 5% wasted, and 17% underweight [6]. Besides, undernutrition plays a role in almost half of the deaths of CU5 worldwide [2]. It increases healthcare expenditure, reduces productivity and slows economic growth [2]. In an African country such as Ghana, child malnutrition costs USD 2.60 billion, or 6.40% of the Gross Domestic Product (GDP) [7].

In response to the burdens caused by child malnutrition, a range of initiatives and commitments have been made by States at the international level to guide strategies to combat it. These include the 2030 Sustainable Development Agenda via its goal 2.2 [2], the United Nations Decade of Action for Nutrition 2016–2025 [2], and the Scaling Up Nutrition (SUN) Movement [8], etc. At the national level in Benin, Goal 1.4 of the National Development Plan 2018–2025 aims to “ensure food and nutritional security and access to safe drinking water for all” [9]. Strengthening research on the main drivers of child malnutrition has emerged as a strategy to promote child survival and development. Indeed, knowledge of the characteristics that constitute risk factors or, on the other hand, protective factors against forms of malnutrition among CU5 is necessary to adopt strategies and implement interventions. Based on studies conducted in different settings, there is consensus that some factors are drivers of children’s healthy nutritional status. The United Nations Children’s Fund (UNICEF) conceptual framework on malnutrition describes immediate, underlying and fundamental causes of malnutrition [10]. Also, some studies highlight the role of child, maternal, and household characteristics in addition to environmental factors [11–13].

Specifically, concerning household factors, the extent to which the Water, Sanitation and Hygiene (WASH) conditions in which CU5 live determine their nutritional status is not agreed upon and is still being discussed. Access to water is sufficient, affordable and physically accessible coverage with safe water of acceptable quality. Access to sanitation refers to the availability and use of facilities or services to dispose of urine and faeces. Hygiene refers to conditions and practices at the individual or community level to prevent infection through contamination. Concerning the relationship between WASH conditions and the nutritional status of CU5, there is a close symmetry between the maps showing the level of WASH coverage and the prevalence of forms of child malnutrition [4, 14]. That is, regions with a high prevalence of stunting, wasting or underweight (Africa and Asia) among CU5 are also those where people have the least access to adequate WASH services [4, 14]. Indeed, notwithstanding the progress made over the past two decades, current WASH coverage remains insufficient, particularly in low- and middle-income countries (Africa and Asia), which have the highest prevalence of child malnutrition. In 2020, 489 million people (7% of the world’s population) still lacked access to improved water services, including 122 million people (2% of the world’s population) using surface water, notably in Africa and Asia [14]. In addition, 1.69 billion people (14% of the world’s population) were not using improved sanitation facilities, with 494 million people (6% of the world’s population) practising open defecation, particularly in Africa and Asia [14]. About 670 million people lack hygiene facilities (soap and water), notably in Africa and Asia [14]. In addition, analytical data show the protective effects of WASH services on the odds/risk of child malnutrition, both at the household and community levels [13, 15–23]. However, other studies did not find evidence of any significant effect of household coverage with adequate individual WASH facilities on reducing the odds of stunting, wasting, or underweight in CU5, after adjusting for confounders [24–28]. Thus, there are mixed findings on the contribution of WASH facilities in the child’s immediate (household) environment to nutritional status.

In Benin, the issue of people’s access to WASH is more relevant than ever. About 6% and 54% of households used surface water as drinking water and practised open defecation, respectively [29]. About 45% of households do not have a place to wash their hands [29]. Added to these inadequate levels of access to WASH services are disparities according to household characteristics [29]. Evidence about the contribution of access to WASH services on the indicators of nutritional status is scarce in Benin. This work aimed to study the effects of household WASH conditions to which CU5 are exposed on their nutritional status in Benin.

## Methods

### Study setting

Benin is a state of 114,763 km² located in West Africa [30]. It has 12 departments, themselves divided into 77 communes [30, 31]. In 2021, Benin’s population was estimated at 12.53 million, including about 2.09 million children under age five (51% boys and 49% girls) [32]. Under-five mortality is estimated at 86 per 1,000 live births [33].

Benin’s national health system has a pyramid structure based on territorial divisions and consists of three levels: central, intermediate, and peripheral. The Ministry of Health manages the central level, whose mission is to design and monitor the state’s health policy [34]. At this level are reference healthcare structures such as the National Hospital and University Centre Hubert Koutoukou Maga [32]. Numbering 12, the Departmental Health Directorates administer the intermediate level [32]. They are responsible for implementing the health policy defined by the government, and planning and coordinating all health service activities in the periphery [35]. At the intermediate level, there are also Departmental University Hospitals, which serve as reference structures for cases coming from the peripheral level [32]. The peripheral level is the base of the health pyramid and includes 34 health zones spread throughout the national territory, each administered by the health zone coordination office [32]. The health zone is organized as a network of first-contact public and private health facilities, all supported by a first-reference hospital to serve between 100,000 and 200,000 inhabitants [32].

### Study type and data source

The study utilized a cross-sectional design and consisted of secondary analyses using datasets from the fifth Demographic and Health Survey (DHS-V) conducted in Benin from 6 to 2017 to 28 February 2018.

### Study population

The study population consisted of successfully surveyed children born five years before DHS-V. Children who died, did not usually reside in the surveyed households or for whom data were missing for the variables of interest were excluded. In addition, for children born of multiple pregnancies, we retained the “first twin” and excluded the others.

### Overview of the DHS-V sampling

**Fig. 1:**
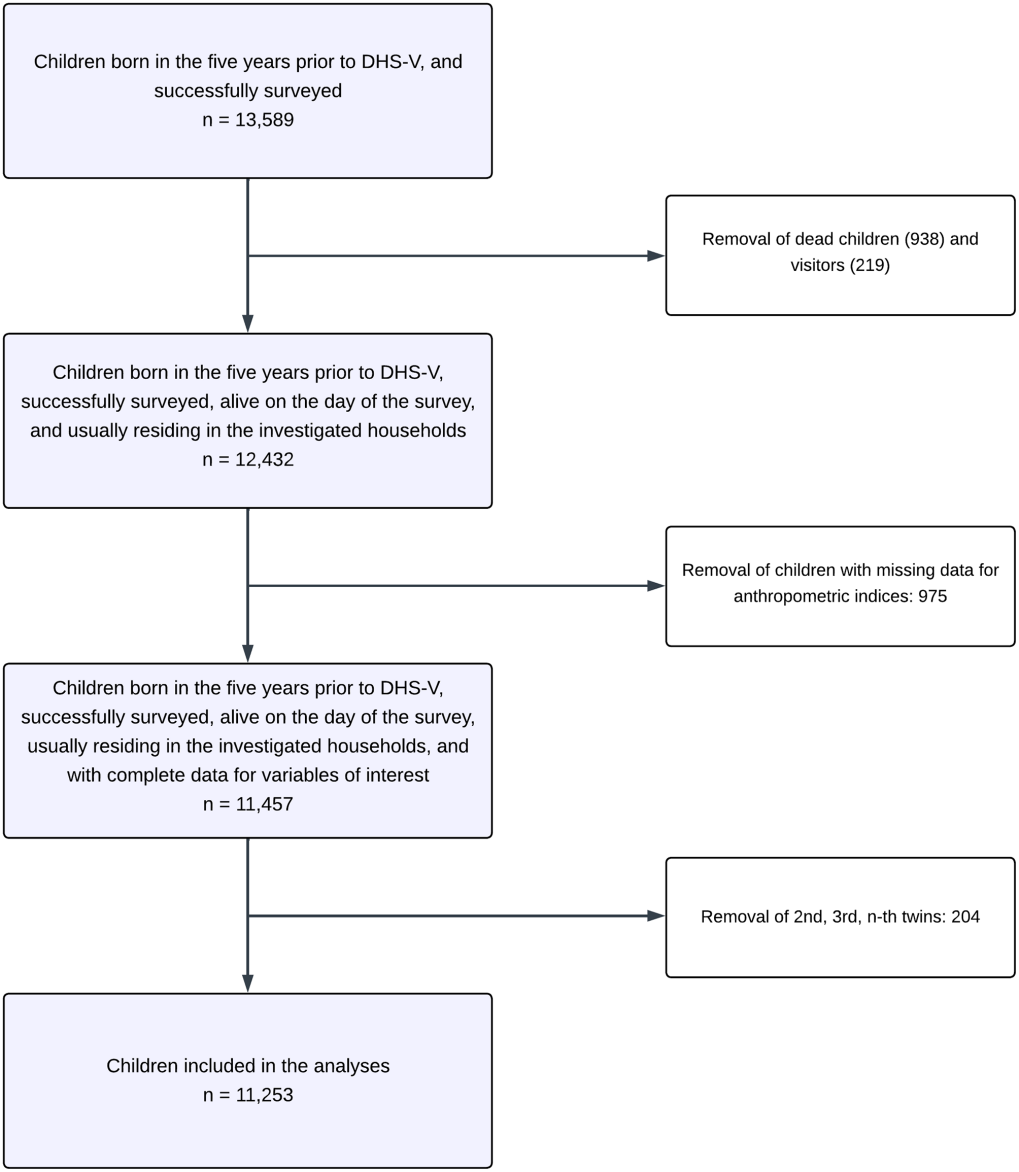
Flow-chart of the children included in the final analysis

The DHS-V was based on a stratified two-stage random sampling to have a nationally representative sample of the Beninese population [6]. The national territory was divided into 23 strata by dividing each department into urban and rural, except the Littoral, which was entirely urban [6]. In each stratum, a specific number of Primary Survey Units (PSUs) were selected in the first degree through a systematic draw with a probability proportional to size (555 PSUs in total), using the list of Enumeration Areas (EAs) established from the Fourth General Census of Population and Housing (RGPH-IV) [6]. In the second stage, after a count, a systematic sample of 26 households was drawn from each PSU [6]. Overall, 14,435 households were successfully surveyed [6]. All children usually living in the surveyed households were eligible to be interviewed. Details on the survey sampling are available elsewhere [6]. The study included 11,253 CU5 (Fig. 1).

### Variables and measures

#### Dependent variables

Three dependent variables were studied, namely stunting, wasting and underweight, coded “1” if the child was stunted, wasted and underweight, respectively, and 0 otherwise. The DHS-V collected data on nutritional status by asking for information on age and then measuring the weight and height of CU5 [6]. Weight was measured with a double-weighing electronic scale (SECA type) and height with a graduated scale (ShorrBoards®) [6]. Children who were younger than two years old were measured while lying down on the scale, whereas those who were older than two years were measured while standing up. The age information of the children was obtained either from official documents or calculated based on the special events calendar [6]. Data on height, weight and age were used to calculate three indices (height-for-age, weight-for-height and weight-for-age). Each index was expressed as a z-score that indicates whether a child’s anthropometric measurements deviate from the norm (World Health Organization Child Growth Standards 2006) [36, 37]. Children whose height-for-age z-score, weight-for-height z-score, and weight-for-age z-score were less than two standard deviations from the median of the reference population suffer from stunting, wasting and underweight, respectively [36, 37].

#### Main exposures

These were the WASH conditions in which CU5 lived in their immediate environment, i.e. at the level of their households. These were grouped as ‘basic’ (at least basic), ‘limited’, ‘unimproved’ and ‘no service’, respectively, according to the WHO/UNICEF Joint Monitoring Programme (JMP) guidelines [14]. The operating definitions of the WHO/UNICEF JMP Scale for WASH services are in Additional File 1.

#### Covariates

The covariates were related to the child, mother (caregiver), household and environment. The child-related covariates include: age in months, sex, rank, twin and diarrhoea in the two weeks before the survey. Birth weight was not studied due to missing data (60%). The children’s mother-related covariates were: age in years, level of education, marital status, professional activity in the last 12 months, health insurance coverage and media exposure, including newspapers, radio or television. The household-related covariates include: sex of the household head, wealth index and household size. The environment-related covariates were: area and department. Data were provided by mothers or caregivers or by observation. The categories of the covariates, including the reference category, are presented in Additional File 1.

### Data analysis

Variables were described by the weighted percentages of their categories. The prevalence of stunting, wasting and underweight were estimated with their 95% confidence intervals (95% CI). Multivariate logistic regressions were performed to assess the effects of individual WASH conditions in which CU5 live on stunting, wasting and underweight, adjusted for covariates. Previously, covariates were selected using univariate analysis by simple logistic regression (p < 0.20) [38]. Following a stepwise backward strategy, the covariates selected from the univariate analysis and the individual WASH-related variables were included in each of the three initial models. Then, the least significant covariates were removed one by one until the remaining ones were all significant in each of the three models. Variables related to WASH conditions were retained in each of the three final models regardless of their significance level. The relationships between the WASH conditions to which CU5 are exposed and the dependent variables were presented with crude (univariate analysis) and adjusted (multivariate analysis) Odds Ratios (OR) and 95% confidence intervals (95% CI). The Hosmer-Lemeshow test was used to evaluate the final models’ goodness of fit. Data analysis was performed with Stata 15 and took into account the survey’s sampling design.

### Ethical aspects

This study was undertaken following the guidelines outlined in the Declaration of Helsinki. The DHS-V has received a statistical visa from the National Statistics Council and a favourable ethical opinion from the National Ethics Committee for Health Research in Benin [6]. The ICF Ethics Committee also evaluated and approved the survey protocol [6]. Before starting the interviews, eligible respondents (mothers or caregivers) gave informed consent [6]. Anthropometric measurements in children were taken after obtaining authorization from the mother or caregivers. More information on the ethical aspects is available elsewhere [6].

## Results

### Basic characteristics of the study population

Table 1 presents the basic characteristics of CU5 in Benin. Children aged 12 months and over were the most represented. There were 50.84% boys and 49.16% girls. Most children (58.95%) were of birth rank three and above. Almost 3% of the children were twins. More than 10% of the children had at least one episode of diarrhoea in the two weeks preceding the survey. The majority of the children’s mothers were from 20 to 29 years old (51.49%), uneducated (65.06%) and in a couple (94.35%). More than eight out of ten mothers (83.34%) had a job in the 12 months before the survey. The children’s mothers were predominantly Christian (50.43%) and Muslim (33.24%). Less than one per cent (0.86%) of children’s mothers reported coverage by health insurance. Also, 2.25%, 34.96% and 18.02% of the children’s mothers read newspapers, listened to the radio and watched television at least once a week, respectively. Most CU5 lived in male-headed households (84.39%), with more than five people (61.86%). We note that 20.53% of children were in the poorest households, while 18.57% belonged to the richest households. The majority of children lived in rural areas (60.64%). Children from Alibori (13.63%), Borgou (12.61%) and Atlantique (11.29%) were the most represented.

**Table 1:** Basic characteristics of children under five in Benin, 2017–2018

Variables	n	%
**Child’s age (months)**		
< 06	1,304	11.56
06–11	1,419	12.58
12–23	2,329	20.63
24–35	2,117	18.76
36–47	2,112	18.72
48–59	2,004	17.76
**Child’s sex**		
Male	5,738	50.84
Female	5,548	49.16
**Child’s rank**		
1	2,419	21.44
2	2,213	19.61
3 and above	6,653	58.95
**Twin**		
No	10,991	97.39
Yes	294	2.61
**Child’s diarrhoea**		
Non	10,068	89.21
Oui	1,218	10.79
**Mother’s age**		
15–19	486	4.31
20–29	5,811	51.49
30–39	4,054	35.93
40–49	935	8.28
**Mother’s level of education**		
No-formal education	7,343	65.06
Primary	2,081	18.44
Secondary	1,712	15.17
Higher	150	1.33
**Mother’s marital status**		
Single	637	5.65
In couple	10,649	94.35
**Mother’s professional activity**		
No	1,880	16.66
Yes	9,405	83.34
**Mother’s religion**		
Traditional and other	1,214	10.75
Islam	3,751	33.24
Christianity	5,691	50.43
No religion	630	5.58
**Mother’s health insurance**		
No	11,189	99.14
Yes	97	0.86
**Mother’s exposure to newspapers**		
Not at all	10,663	94.48
Less than once a week	369	3.27
At least once a week	254	2.25
**Mother’s exposure to radio**		
Not at all	4,959	43.94
Less than once a week	2,382	21.11
At least once a week	3,945	34.96
**Mother’s exposure to television**		
Not at all	7,404	65.60
Less than once a week	1,848	16.38
At least once a week	2,034	18.02
**Household head’s sex**		
Male	9,524	84.39
Female	1,762	15.61
**Household wealth index**		
Poorest	2,317	20.53
Poorer	2,256	19.99
Middle	2,308	20.45
Richer	2,309	20.46
Richest	2,096	18.57
**Household size**		
≤ 5	4,304	38.14
> 5	6,982	61.86
**Area**		
Urban	4,442	39.36
Rural	6,844	60.64
**Department**		
Alibori	1,538	13.63
Atacora	1,001	8.87
Atlantique	1,274	11.29
Borgou	1,423	12.61
Collines	744	6.59
Couffo	751	6.66
Donga	742	6.58
Littoral	504	4.47
Mono	513	4.54
Ouémé	1,023	9.06
Plateau	688	6.09
Zou	1,085	9.62

### Access to WASH services

**Fig. 2:**
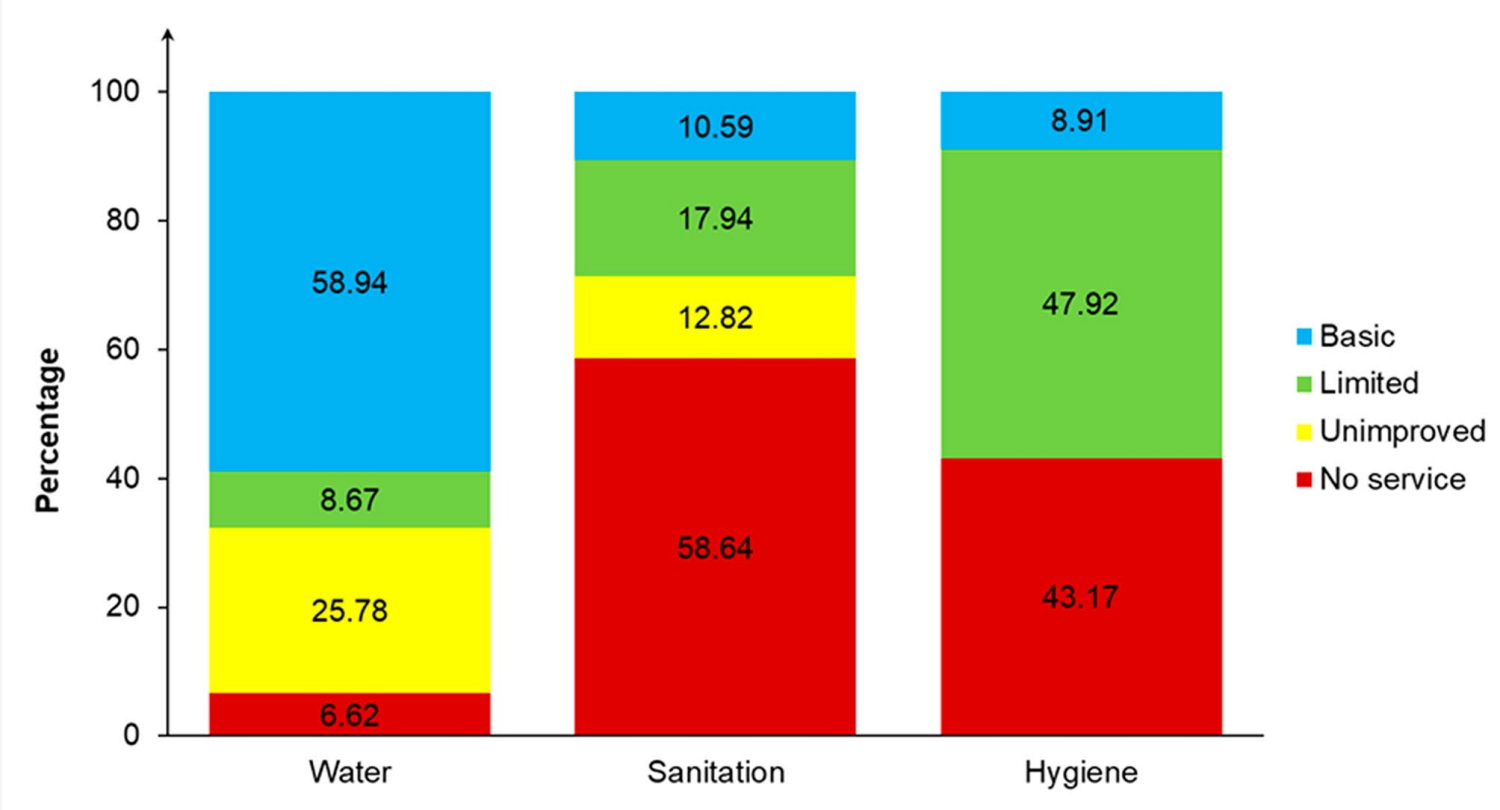
Distribution of CU5 by household access to WASH services in Benin, 2017–2018

Figure 2 shows the distribution of surveyed children by level of access to individual WASH services in their households. About 60% (95% CI = 56.28–61.54) of CU5 lived in households with access to basic water services, compared to 6.62% (95% CI = 5.22–8.35) in ones using surface water. Of the children, 58.64% (95% CI = 55.70-61.52) lived in households without toilets. One in ten (10.59%, 95% CI = 9.33–12.01) children were in households using unshared improved toilets (basic). Another 43.17% (95% CI = 40.80-45.56) of children were from households without hygiene facilities.

### Prevalence of malnutrition

**Fig. 3:**
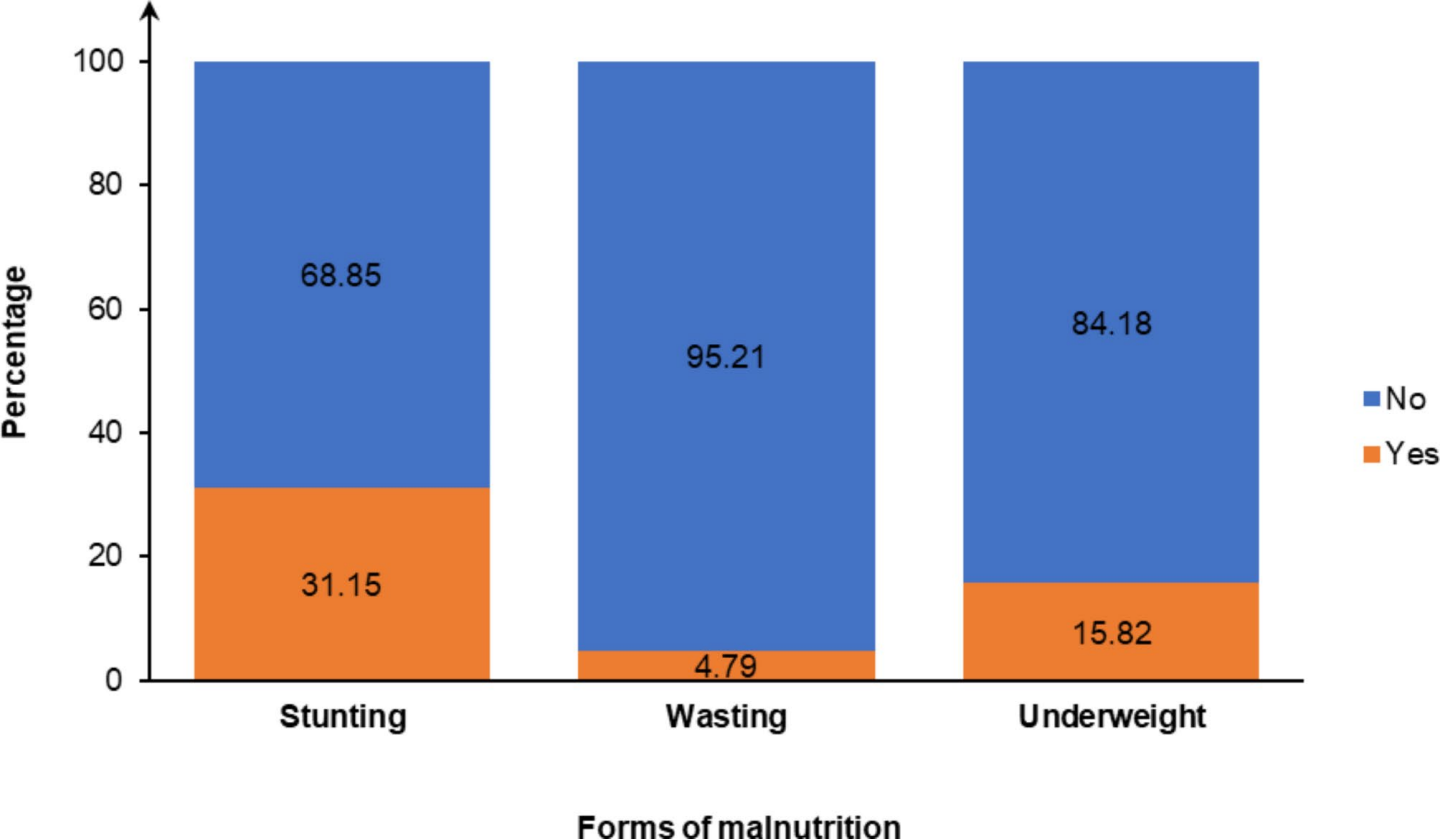
Prevalence of stunting, wasting and underweight among CU5 in Benin, 2017–2018

The prevalence of stunting, wasting, and underweight in CU5 was 31.15% (95% CI = 29.90-32.42), 4.79% (95% CI = 4.33–5.31), and 15.82% (95% CI = 14.92–16.76), respectively (Fig. 3).

### Effects of WASH conditions on malnutrition in CU5

**Fig. 4:**
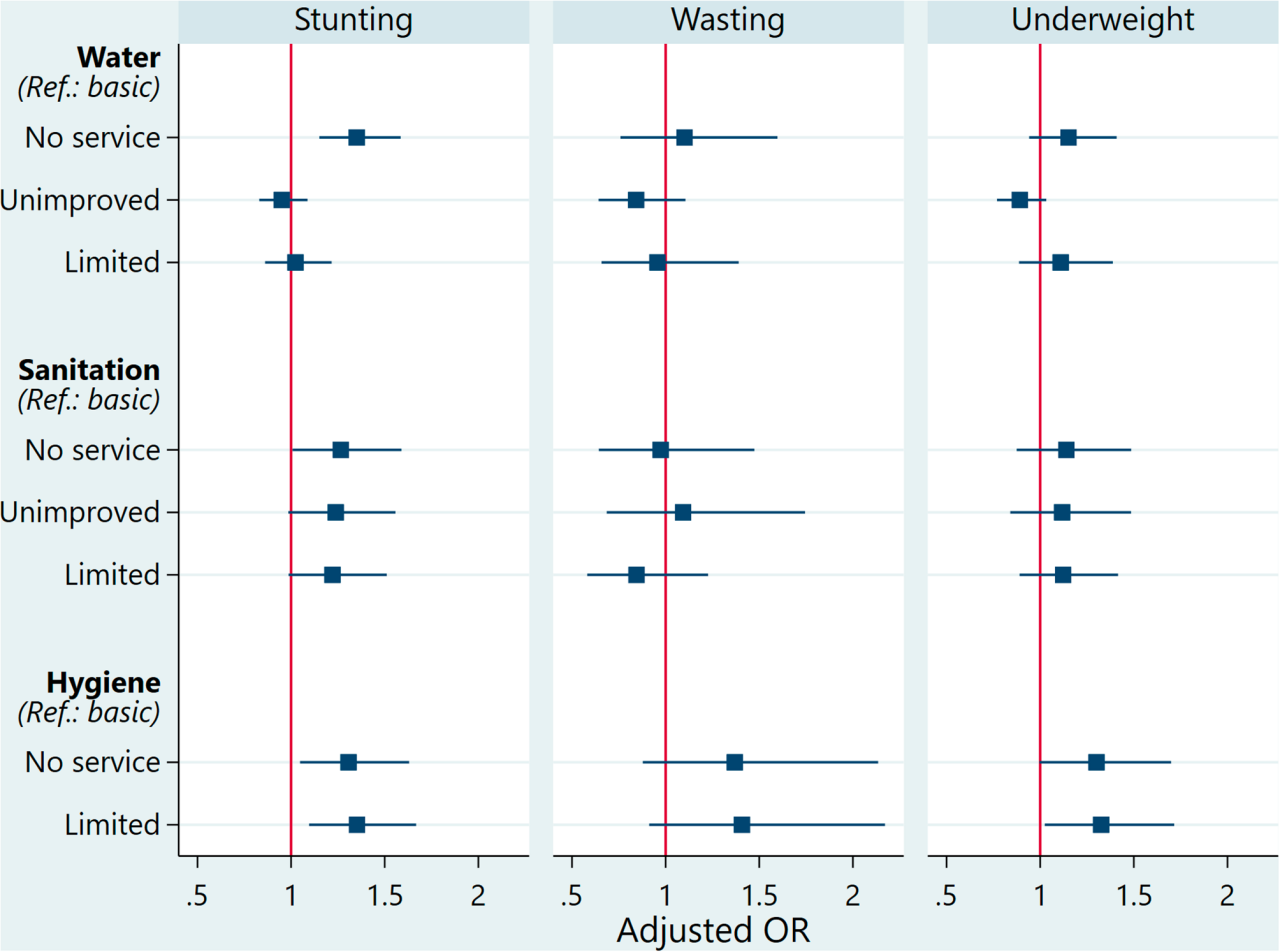
Effects of WASH conditions on malnutrition in CU5, Benin, 2017–2018

Figure 4 summarises the effects of WASH conditions in which CU5 live on stunting, wasting and underweight, adjusted for confounders. The full regression results are set out in Additional File 2. The odds of stunting for children from households using surface water were 1.35 (95% CI = 1.15–1.59) times higher than for children living in households with basic water services. CU5 living in households practising open defecation were 1.27 (95% CI = 1.01–1.59) times more likely to be stunted than children in households with basic sanitation. CU5 in households with no hygiene facilities and those using limited hygiene services were 1.31 (95% CI = 1.05–1.63) and 1.35 (95% CI = 1.10–1.67) times more likely to be stunted, respectively, compared to children in households with basic hygiene facilities. After adjusting for confounders, household access to water and sanitation lost its significance to underweight. The type of hygiene facility remained significant. The odds of being underweight were 1.33 (95% CI = 1.02–1.72) times higher among children under five from households with limited hygiene facilities than among children from households with basic hygiene facilities. After adjusting for child, mother, household, and environmental characteristics, there was no significant relationship between WASH facilities and wasting among children.

## Discussion

This study investigated whether the WASH conditions faced by CU5 affect their nutritional status. Stunting is a sign of chronic malnutrition, resulting from inadequate nutrition over a long time or from recurrent and chronic illnesses [6]. The study found that 31.15% of the CU5 suffered from stunting, which is about 10% lower than in 2006 [39]. One study (2020) based on national data from 35 low- and middle-income countries found that the prevalence of stunting among children aged 12–59 months was 38.8% [13]. Furthermore, the prevalence of stunting among CU5 in Benin found in the current study is similar to that estimated for West Africa (31.8%, 95% CI = 28.1–35.5), Southern Africa (30.6%, 95% CI = 22.4–38.9) and Central Africa (28.8%, 95% CI = 21.4–36.2) in a 2017 study [5]. Specifically, in the West African sub-region, the prevalence of stunting among CU5 ranged from 18.8% in Ghana to 43.9% in Niger [5]. The prevalence of stunting in East Africa was higher as almost 40% of children were affected (39.0%, 95% CI = 33.6–44.4) [5]. Elsewhere in Africa, studies report a prevalence of 38.4% in Ethiopia (2020) and 40% in Zambia (2018) [15, 28]. In Asia, a prevalence ranging from 25 to 50% was found in India (2015), Indonesia (2016–2017) and Nepal (2020) among children [19, 23, 26, 27]. In Papua New Guinea, more than half of CU5 were stunted in 2012 [40].

Wasting is a measure of acute malnutrition and the result of inadequate food intake or an episode that has resulted in weight loss over a recent period [6]. In 2006, the prevalence of wasting among CU5 in Benin was 8% [39]. The present study found that 4.79% of children suffered from wasting. This proportion is less than half that estimated for West Africa (10.0% vs. 4.79%) [5]. It remains similar to the prevalence found in East Africa (5.4%, 95% CI = 4.4–6.5), Southern Africa (4.1%, 95% CI = 2.1–6.2) and Central Africa (6.7%, 95% CI = 4.2–9.2) in a 2017 study [5]. Notably, in the West African sub-region, wasting prevalence among CU5 ranged from 4.7% in Ghana to 18.0% in Niger [5]. In Ethiopia (2020), 10% of CU5 were wasted [28]. In Asia, a higher prevalence of wasting was found in Nepal (10%) [23] and India (16–27%) among children [19]. In Papua New Guinea, 18% of CU5 were wasted in 2012 [40]. Among children aged 12–59 months, a prevalence of 12.9% was found in low- and middle-income countries in a 2020 study [13].

Underweight encompasses both acute and chronic undernutrition and is an indicator of overall malnutrition [6]. In 2006, the prevalence of underweight among CU5 in Benin was 18% [39]. The current study reported that 15.82% of the children were underweight. This finding is lower than that calculated for the West African region (20.1%, 95% CI = 15.9–24.4) in 2017 [5]. The prevalence of underweight among CU5 in Eastern, Southern and Central Africa was 14.4% (95% CI = 9.5–19.6), 10.7% (95% CI = 4.8–16.5) and 12.8% (95% CI = 6.7–18.9), respectively [5]. In Ethiopia (2020), 23.73% of CU5 were underweight [28]. Within the West African sub-region, the prevalence of underweight in CU5 ranged from 11.0% in Ghana to 36.4% in Niger [5]. In Nepal (2020), 27% of children were underweight [23]. In Papua New Guinea (2012), almost one-third of CU5 were underweight [40]. In low- and middle-income countries (2020), the prevalence of underweight was 27.5% among children aged 12–59 months [13].

The differences highlighted regarding the prevalence of stunting, wasting, and underweight between our work and those conducted in other geographic areas would be linked to the variability of socio-demographic, economic, political, health, and other contexts that favour the occurrence of these conditions in CU5.

Nearly 60% of CU5 lived in households with access to basic water services. Also, 8.67% were covered by limited facilities. Thus 67.61% of CU5 lived in households with improved water services. Ethiopia (2020) recorded lower coverage (56.43%) [28]. Other studies have found higher coverage of improved water facilities among children [19, 25, 27]. In the current study, the coverage of CU5 with basic sanitation facilities was 10.59%. In Ethiopia (2016), 7.46% of CU5 lived in households with basic sanitation facilities [41]. In Indonesia (2017), more than two-thirds of children came from households covered by basic sanitation services [27]. In addition, the current study showed that 17.94% of CU5 lived in households with improved toilets but shared by others. Thus, 28.53% of children lived in households with improved toilets. In Ethiopia (2020), a national survey found that 9.94% of CU5 had access to improved sanitation facilities [28]. A much higher coverage (44.13%) was observed in northern Ethiopia in 2021 [18]. In Burkina Faso (2017), about 24% of CU5 lived in households with improved toilets [25]. In India, one-fifth of CU5 were in households using improved sanitation facilities in 2015 [19]. A national survey estimated that 75.3% of CU5 lived in households with improved toilets in Nepal [23]. According to the present study, only 8.91% of children lived in households with basic hygiene facilities. Higher proportions, from 37.5 to 67.9%, were found in Indonesia (2016–2017), Ethiopia (2020) and Nepal (2020) [23, 26–28].

Our study found an association between the level of access to water sources and chronic malnutrition (stunting) among CU5 after adjustment for confounders. Children in households using surface water had 1.35 times higher odds of experiencing stunting than those in households with basic water services. Similar to our findings, some papers have found a significant negative relationship between household access to improved water sources and the prevalence of stunting among CU5. A 2011 study found that children from households with access to a high-quality water source have 9% lower odds of being stunted than those with low-quality water facilities [16]. Also, a cluster randomised controlled trial in 2012 estimated a 12.1% reduction in the prevalence of stunting after an intervention to increase access to adequate WASH services [17]. In Ethiopia (2021), the odds of stunting for CU5 were twice as high if they lived in households without access to improved water facilities [18]. In contrast, some studies have found no significant effect of household access to improved water facilities on the risk of stunting in CU5 [13, 19, 20, 24, 25, 27, 28]. The reliability or quality of water, even from an improved or basic source, can be affected by various unhygienic behaviours [28].

Concerning access to improved sanitation facilities, this study found a negative relationship between household access to sanitation facilities and stunting in CU5. The stunting odds were 1.27 times higher for children from households with no sanitation services than for children living in households with basic sanitation services. Other studies reported similar findings [13, 16, 18–20, 23]. Overall, in low- and middle-income countries, access to improved toilets was associated with a 10% reduction in the stunting odds among children aged 12–59 months [13]. In Ethiopia (2016), the odds of stunting were 1.26 times higher among children living in households with unimproved latrines [41]. In India (2015), the reduction in stunting odds associated with household access to improved sanitation facilities was 16% [19]. However, some studies have not found a significant relationship between the level of household access to sanitation and stunting [24–28].

According to the current study, access to basic hygiene facilities was a protective factor against stunting in CU5. Studies in Ethiopia (2021), India (2015) and Nepal (2020) found a significant negative relationship between household access to handwashing facilities with soap and water and stunting in CU5 [18, 19, 23]. In contrast, some studies have found no significant relationship between these two variables [27, 28]. The present study did not explore possible interactions between the exposure variables. In their association with child stunting, some work observed an interaction of sanitation conditions and hygiene practices with household access to water services [19]. In India (2015), access to improved toilets (-16%) and hygiene (-14%) facilities were protective factors against stunting in children. However, the protective effects of these facilities were more pronounced (-23%, respectively) in households with access to piped water [19]. In another study (2020), children who had combined access to improved sanitation and hygiene facilities were 29% less likely to be stunted [28]. No significant effect was observed with individual coverage in these facilities [28]. Also, children living in households with combined access to improved WASH facilities were 33% less likely to be stunted, with no significant effect under the coverage of individual WASH facilities [28]. One study (2020) found that the gain associated with water purification was higher in rural than urban areas [23].

The level of household access to water services did not significantly affect wasting in the surveyed children. Similarly, in Ethiopia (2019), household access to improved water sources was not associated with a reduction of wasting among children aged 6–59 months [22]. In our study, the relationship between wasting and water source purification was not investigated. A 2020 study in Nepal noted that home water purification was associated with a decrease in wasting odds [23]. However, water purification was not associated with stunting prevention. So, its effect seemed either offset for more severe undernutrition forms or that children experience catch-up growth without a significant impact on long-term nutritional health [23].

In opposition to the present study, a 2020 study involving 35 low- and middle-income countries found a negative association between acute malnutrition and household access to sanitation facilities [13]. Also, a 2019 study in Ethiopia showed a negative association between acute malnutrition and household access to sanitation facilities [22]. Children living in households with access to improved toilets were 37% less likely to be wasted than children from ones using unimproved toilets [22]. A similar observation was made in Nepal (2020) [23]. We also did not note an association between household access to hygiene services and wasting.

As such, we observe an influence of WASH conditions faced by CU5 on stunting, but not on wasting. It appears that the detrimental effects of insufficient WASH conditions primarily manifest over a long time, impacting the growth and development of children, with little or no immediate effect on their short-term nutritional status. However, further research is needed to better understand the underlying mechanisms and complex interactions between WASH conditions and the nutrition of CU5.

In general, few studies have examined the effects of WASH conditions faced by CU5 on underweight, compared to stunting and wasting. In the present study, the level of access to water and sanitation did not significantly affect underweight in CU5. Data from low- and middle-income countries suggest that CU5 living in households with access to improved sanitation facilities were less likely to be underweight [13]. In our study, CU5 covered with limited hygiene facilities were 1.33 times more likely to be underweight than children in ones with basic hygiene facilities. In Nepal (2020), hygiene practice resulted in a 41% reduction in the odds of being underweight [23]. In Ethiopia (2020), children with access to improved handwashing facilities were 17% less likely to be underweight than those with access to unimproved facilities [28].

We end the discussion of the study’s results by mentioning its strengths and limitations. The use of nationally representative data, which contributes to the generalisation of results, is one of the strengths of this study. Also, this is one of the first studies in Benin to explore the influence of inadequate WASH conditions to which children are exposed on their nutritional status. There are some limitations to the results of the study. A causal relationship between exposures and outcome variables is not clear due to the cross-sectional design of the study. Another limitation is the lack of behavioural and nutritional variables. It is mainly due to their absence in the datasets or the critical percentage of missing data. In addition, some variables were only available in a sub-sample.

## Conclusion

This study contributes to the growing literature on the relationship between the level of household access to WASH facilities and the nutritional status of CU5. The effects of WASH services varied across the forms of child malnutrition. Stunting was significantly associated with household access to individual WASH services. But wasting was not. Also, CU5 from households using inadequate hygiene facilities were more likely to be underweight. Interventions to fight malnutrition in CU5 should include a WASH dimension. Further studies with robust designs and sufficient power could analyse the interactions between WASH facilities to quantify their synergistic effect on child nutrition.

## Electronic supplementary material

Below is the link to the electronic supplementary material.

Additional File 1

Additional File 2

## Data Availability

The data that support our findings are available by sending a request to the DHS program via https://dhsprogram.com/. Before downloading datasets, you must first register as a DHS data user. Access to datasets is granted for research purposes only.

## Abbreviations

95% CI: 95% Confidence Interval
CU5: Children Under Five
DHS: Demographic and Health Survey
DHS-V: Fifth Demographic and Health Survey
EA: Enumeration Area
GDP: Gross Domestic Product
JMP: Joint Monitoring Programme
OR: Odds Ratios
p: p-value
PSU: Primary Survey Unit
RGPH-IV: Fourth General Census of Population and Housing
SUN: Scaling Up Nutrition
UNICEF: United Nations Children’s Fund
USD: United States Dollar
WASH: Water, Sanitation and Hygiene
WHO: World Health Organization
