# Supplementary material for: Effects of the level of household access to water, sanitation and hygiene on the nutritional status of children under five, Benin
Source: BMC Nutr. 2023 Aug 1;9:95. doi: 10.1186/s40795-023-00751-8 (PMC10391820; doi:10.1186/s40795-023-00751-8)
Supplement: Supplementary file 1 — Additional File 1 [file 40795_2023_751_MOESM1_ESM.pdf]

List of tables

Table A1. WHO/UNICEF JMP scale for WASH services. 2
Table A2. JMP classification of improved/unimproved water and sanitation facility types 3
Table A3. Presentation of the studied covariates 4

Table A1. WHO/UNICEF JMP scale for WASH services. Adapted from WHO, UNICEF. Progress on household drinking water, sanitation and hygiene 2000-2020: five years into the SDGs. Geneva, Switzerlanda: World Health Organization; 2021.

Service ladder	Water	Sanitation	Hygiene
Basic (at least basic)	Improved water point providing drinking water, where the round trip to collect water does not exceed 30 minutes, including waiting time	Improved facility not shared with other households	Hand hygiene facility with soap and water available at home
Limited	Improved water point providing drinking water, where the round trip to collect water exceeds 30 minutes, including waiting time	Improved facility shared with other households	Handwashing facility without water or soap available at home
Unimproved	Water point from an unprotected well or unprotected spring	Flush and pour-flush toilets flushed to an open drain or elsewhere, pit latrines without slabs, open pits, hanging toilets/latrines, bucket latrines, including pans, trays or other unsealed containers	Not applicable
No service	Surface water: water directly from a river, dam, lake, pond, stream, canal or irrigation canal	Open defecation	No handwashing facility available in the home

Table A2. JMP classification of improved/unimproved water and sanitation facility types. Adapted from WHO, UNICEF. Progress on household drinking water, sanitation and hygiene 2000-2020: five years into the SDGs. Geneva, Switzerlanda: World Health Organization; 2021.

Service ladder	Water	Sanitation
Improved facilities	Piped supplies<br>• Piped supplies<br>• Tap water in the dwelling, yard or<br>• plot, including piped to a neighbour<br>• Public taps or standpipes<br>• Non-piped supplies<br>• Boreholes/tubewells<br>• Protected wells and springs<br>• Rainwater<br>• Packaged water, including bottled water and sachet water<br>• Delivered water, including tanker trucks and small carts/tanks/drums<br>• Water kiosks	Networked sanitation<br>• Flush and pour-flush toilets connected to sewers<br>• On-site sanitation<br>• Flush and pour-flush toilets or latrines connected to septic tanks or pits<br>• Ventilated improved pit (VIP) latrines<br>• Pit latrines with slabs (constructed from materials that are durable and easy to clean)<br>• Composting toilets, including twin pit latrines with slabs and container-based systems
Unimproved facilities	Non-piped supplies<br>• Unprotected wells and springs	Networked sanitation<br>• Flush and pour-flush toilets flushed to an open drain or elsewhere<br><br>On-site sanitation<br>• Pit latrines without slabs<br>• Open pits<br>• Hanging toilets/latrines<br>• Bucket latrines, including pans, trays or other unsealed containers

Table A3. Presentation of the studied covariates

Variables	Categories	Reference
**Child's age (months)**	<06	✗
	06-11	
	12-23	
	24-35	
	36-47	
	48-59	
**Child's sex**	Male	
	Female	✗
**Child's rank**	1	
	2	
	3+	✗
**Twin**	Yes	
	No	✗
**Child's diarrhoea**	Yes	
	No	✗
**Mother's age**	15-19	
	20-29	
	30-39	
	40-49	✗
**Mother's level of education**	No-formal education	
	Primary	
	Secondary	
	Higher	✗
**Mother's marital status**	Single	
	In couple	✗
**Mother's professional activity**	Yes	✗
	No	
**Mother's religion**	Christians	✗
	Traditional and other	
	Islam	
	No religion	
**Mother's health insurance**	Yes	✗
	No	
**Mother's exposure to newspapers**	Not at all	
	Less than once a week	
	At least once a week	✗

Table A3. continued

Variables	Categories	Reference
**Mother's exposure to radio**	Not at all	
	Less than once a week	
	At least once a week	✗
**Mother's exposure to television**	Not at all	
	Less than once a week	
	At least once a week	✗
**Household head's sex**	Male	✗
	Female	
**Household wealth index**	Poorest	
	Poorer	
	Middle	
	Richer	
	Richest	✗
**Household size**	≤5	✗
	>5	
**Area**	Rural	
	Urban	✗
**Department**	Alibori	
	Atacora	
	Atlantique	
	Borgou	
	Collines	✗
	Couffo	
	Donga	
	Littoral	
	Mono	
	Ouémé	
	Plateau	
	Zou	
