# Supplementary material for: Effects of the level of household access to water, sanitation and hygiene on the nutritional status of children under five, Benin
Source: BMC Nutr. 2023 Aug 1;9:95. doi: 10.1186/s40795-023-00751-8 (PMC10391820; doi:10.1186/s40795-023-00751-8)
Supplement: Supplementary file 2 — Additional File 2 [file 40795_2023_751_MOESM2_ESM.pdf]

List of acronyms and abbreviations

95% CI	:	95% confidence intervals
aOR	:	adjusted odds ratios
cOR	:	crude odds ratios
p	:	p-value

List of tables

Table B1. Factors associated with stunting in children under five, Benin, 2017-2018 2
Table B2. Factors associated with wasting in children under five, Benin, 2017-2018 5
Table B3. Factors associated with underweight in children under five, Benin, 2017-2018 8

Table B1. Factors associated with stunting in children under five, Benin, 2017-2018

Variables	Univariate analysis			Multivariate analysis		
	cOR	95% CI	p	aOR	95% CI	p
**Water**						
No service	1.86	1.60 - 2.17	<0.001	1.35	1.15 - 1.59	<0.001
Unimproved	1.19	1.05 - 1.34	0.008	0.95	0.83 - 1.09	0.459
Limited	1.19	1.00 - 1.40	0.046	1.02	0.86 - 1.22	0.792
Basic	1.00			1.00		
**Sanitation**						
No service	2.32	1.93 - 2.79	<0.001	1.27	1.01 - 1.59	0.043
Unimproved	1.84	1.49 - 2.27	<0.001	1.24	0.99 - 1.56	0.067
Limited	1.47	1.20 - 1.80	<0.001	1.22	0.99 - 1.51	0.066
Basic	1.00			1.00		
**Hygiene**						
No service	1.72	1.40 - 2.12	<0.001	1.31	1.05 - 1.63	0.018
Limited	1.80	1.48 - 2.19	<0.001	1.35	1.10 - 1.67	0.005
Basic	1.00			1.00		
**Child's age (months)**						
<06	1.00			1.00		
6-11	1.38	1.12 - 1.70	0.002	1.39	1.13 - 1.72	0.002
12-23	2.66	2.19 - 3.23	<0.001	2.81	2.30 - 3.42	<0.001
24-35	3.64	3.03 - 4.38	<0.001	4.04	3.33 - 4.90	<0.001
36-47	3.03	2.52 - 3.64	<0.001	3.27	2.71 - 3.95	<0.001
48-59	2.51	2.07 - 3.04	<0.001	2.71	2.23 - 3.29	<0.001
**Child's sex**						
Male	1.29	1.19 - 1.41	<0.001	1.32	1.21 - 1.44	<0.001
Female	1.00			1.00		
**Child's rank**						
1	0.98	0.87 - 1.10	0.709			
2	0.93	0.83 - 1.04	0.231			
3+	1.00					
**Twin**						
Yes	2.76	2.17 - 3.50	<0.001	3.10	2.39 - 4.01	<0.001
No	1.00			1.00		
**Child's diarrhoea**						
Yes	1.23	1.07 - 1.41	0.004	1.29	1.11 - 1.50	0.001
No	1.00			1.00		
**Mother's age**						
15-19	0.92	0.71 - 1.19	0.527			
20-29	0.97	0.82 - 1.15	0.731			
30-39	0.90	0.75 - 1.09	0.284			
40-49	1.00					

Table B1. continued

Variables	Univariate analysis			Multivariate analysis		
	cOR	95% CI	p	aOR	95% CI	p
**Mother's level of education**						
No-formal education	4.93	2.91 - 8.34	<0.001	2.11	1.20 - 3.70	0.010
Primary	3.86	2.26 - 6.58	<0.001	2.09	1.18 - 3.68	0.011
Secondary	2.79	1.65 - 4.71	<0.001	1.83	1.05 - 3.17	0.032
Higer	1.00			1.00		
**Mother's marital status**						
Single	1.07	0.89 - 1.29	0.462			
In couple	1.00					
**Mother's professional activity**						
Yes	1.00					
No	1.02	0.90 - 1.15	0.761			
**Mother's religion**						
Christians	1.00					
Traditional and other	1.43	1.24 - 1.66	<0.001			
Islam	1.15	1.02 - 1.30	0.026			
No religion	1.49	1.21 - 1.84	<0.001			
**Mother's health insurance**						
Yes	1.00					
No	3.49	1.89 - 6.45	<0.001			
**Mother's exposure to newspapers**						
Not at all	2.67	1.78 - 3.99	<0.001			
Less than once a week	1.43	0.89 - 2.29	0.138			
At least once a week	1.00					
**Mother's exposure to radio**						
Not at all	1.37	1.23 - 1.52	<0.001	1.14	1.02 - 1.28	0.022
Less than once a week	1.12	0.98 - 1.27	0.106	1.04	0.90 - 1.20	0.585
At least once a week	1.00			1.00		
**Mother's exposure to television**						
Not at all	1.88	1.65 - 2.16	<0.001			
Less than once a week	1.49	1.25 - 1.76	<0.001			
At least once a week	1.00					
**Household head's sex**						
Male	1.00					
Female	0.94	0.83 - 1.07	0.364			
**Household wealth index**						
Poorest	2.98	2.51 - 3.53	<0.001	2.29	1.76 - 2.98	<0.001
Poorer	2.66	2.24 - 3.17	<0.001	2.21	1.72 - 2.84	<0.001
Middle	2.15	1.80 - 2.56	<0.001	1.80	1.42 - 2.28	<0.001
Richer	1.66	1.39 - 1.99	<0.001	1.45	1.17 - 1.80	0.001
Richest	1.00			1.00		

Table B1. continued

Variables	Univariate analysis			Multivariate analysis		
	cOR	95% CI	p	aOR	95% CI	p
**Household size**						
≤5	1.00					
>5	1.22	1.10 - 1.36	<0.001			
**Area**						
Rural	1.44	1.28 - 1.64	<0.001			
Urban	1.00					
**Department**						
Alibori	1.97	1.51 - 2.56	<0.001	1.94	1.49 - 2.53	<0.001
Atacora	1.87	1.42 - 2.47	<0.001	1.59	1.23 - 2.07	<0.001
Atlantic	1.46	1.11 - 1.93	0.007	1.91	1.48 - 2.46	<0.001
Borgou	1.80	1.37 - 2.36	<0.001	1.74	1.38 - 2.19	<0.001
Collines	1.00			1.00		
Couffo	1.94	1.46 - 2.57	<0.001	2.14	1.64 - 2.80	<0.001
Donga	1.18	0.84 - 1.68	0.336	1.27	0.94 - 1.72	0.123
Littoral	0.79	0.56 - 1.12	0.186	1.69	1.21 - 2.36	0.002
Mono	1.29	0.92 - 1.80	0.140	1.35	0.97 - 1.89	0.074
Ouémé	1.39	1.05 - 1.84	0.022	1.87	1.40 - 2.48	<0.001
Plateau	1.84	1.34 - 2.51	<0.001	1.87	1.40 - 2.50	<0.001
Zou	1.78	1.36 - 2.34	<0.001	2.14	1.65 - 2.76	<0.001

Goodness-of-fit test
F-adjusted test statistic = 0.739
Prob > F = 0.673

Table B2. Factors associated with wasting in children under five, Benin, 2017-2018

Variables	Univariate analysis			Multivariate analysis		
	cOR	95% CI	p	aOR	95% CI	p
**Water**						
No service	1.17	0.83 - 1.66	0.364	1.10	0.76 - 1.60	0.612
Unimproved	1.01	0.80 - 1.29	0.905	0.84	0.64 - 1.11	0.217
Limited	0.89	0.61 - 1.30	0.554	0.96	0.66 - 1.39	0.814
Basic	1.00			1.00		
**Sanitation**						
No service	0.94	0.69 - 1.28	0.683	0.97	0.64 - 1.47	0.899
Unimproved	1.11	0.74 - 1.67	0.605	1.09	0.69 - 1.74	0.707
Limited	0.85	0.60 - 1.19	0.340	0.84	0.58 - 1.23	0.374
Basic	1.00			1.00		
**Hygiene**						
No service	1.36	0.86 - 2.13	0.19	1.37	0.88 - 2.13	0.165
Limited	1.43	0.92 - 2.24	0.12	1.41	0.91 - 2.17	0.122
Basic	1.00			1.00		
**Child's age (months)**						
<06	1.00			1.00		
6-11	1.54	1.14 - 2.09	0.005	1.46	1.07 - 1.98	0.017
12-23	1.14	0.86 - 1.52	0.356	1.12	0.84 - 1.50	0.439
24-35	0.50	0.36 - 0.71	<0.001	0.51	0.36 - 0.72	<0.001
36-47	0.31	0.21 - 0.45	<0.001	0.32	0.22 - 0.47	<0.001
48-59	0.51	0.34 - 0.74	0.001	0.52	0.36 - 0.77	0.001
**Child's sex**						
Male	1.44	1.19 - 1.75	<0.001	1.46	1.20 - 1.77	<0.001
Female	1.00			1.00		
**Child's rank**						
1	1.00	0.78 - 1.29	0.992			
2	1.07	0.85 - 1.36	0.556			
3+	1.00					
**Twin**						
Yes	2.42	1.55 - 3.77	<0.001	2.59	1.65 - 4.06	<0.001
No	1.00			1.00		
**Child's diarrhoea**						
Yes	2.18	1.73 - 2.76	<0.001	1.80	1.43 - 2.27	<0.001
No	1.00			1.00		
**Mother's age**						
15-19	1.81	1.07 - 3.05	0.026			
20-29	1.25	0.87 - 1.79	0.222			
30-39	1.14	0.79 - 1.65	0.480			
40-49	1.00					

Table B2. continued

Variables	Univariate analysis			Multivariate analysis		
	cOR	95% CI	p	aOR	95% CI	p
**Mother's level of education**						
No-formal education	0.88	0.45 - 1.70	0.701			
Primary	0.73	0.37 - 1.44	0.369			
Secondary	0.82	0.43 - 1.58	0.549			
Higher	1.00					
**Mother's marital status**						
Single	1.55	1.08 - 2.22	0.017	1.62	1.12 - 2.33	0.010
In couple	1.00			1.00		
**Mother's professional activity**						
Yes	1.00					
No	1.20	0.96 - 1.51	0.113			
**Mother's religion**						
Christians	1.00					
Traditional and other	0.95	0.68 - 1.32	0.754			
Islam	1.40	1.13 - 1.72	0.002			
No religion	1.00	0.63 - 1.58	0.990			
**Mother's health insurance**						
Yes	1.00					
No	0.57	0.26 - 1.24	0.159			
**Mother's exposure to newspapers**						
Not at all	1.31	0.65 - 2.63	0.452			
Less than once a week	1.42	0.68 - 2.97	0.353			
At least once a week	1.00					
**Mother's exposure to radio**						
Not at all	1.40	1.11 - 1.77	0.005	1.41	1.12 - 1.79	0.004
Less than once a week	0.94	0.71 - 1.25	0.684	0.97	0.73 - 1.29	0.844
At least once a week	1.00			1.00		
**Mother's exposure to television**						
Not at all	0.99	0.74 - 1.34	0.972			
Less than once a week	0.91	0.64 - 1.30	0.607			
At least once a week	1.00					
**Household head's sex**						
Male	1.00					
Female	1.08	0.84 - 1.38	0.544			
**Household wealth index**						
Poorest	0.97	0.71 - 1.34	0.865	0.63	0.40 - 0.99	0.044
Poorer	0.93	0.67 - 1.29	0.670	0.65	0.42 - 1.02	0.061
Middle	0.75	0.53 - 1.06	0.107	0.57	0.37 - 0.87	0.010
Richer	0.89	0.65 - 1.22	0.472	0.74	0.51 - 1.05	0.095
Richest	1.00			1.00		

Table B2. continued

Variables	Univariate analysis			Multivariate analysis		
	cOR	95% CI	p	aOR	95% CI	p
**Household size**						
≤5	1.00					
>5	1.11	0.91 - 1.34	0.305			
**Area**						
Rural	1.01	0.80 - 1.28	0.917			
Urban	1.00					
**Department**						
Alibori	2.17	1.22 - 3.87	0.009	2.46	1.33 - 4.56	0.004
Atacora	1.39	0.76 - 2.54	0.287	1.31	0.74 - 2.34	0.354
Atlantic	1.21	0.67 - 2.18	0.525	1.34	0.74 - 2.41	0.328
Borgou	1.70	0.95 - 3.03	0.074	1.76	1.00 - 3.09	0.048
Collines	1.00			1.00		
Couffo	1.11	0.57 - 2.16	0.754	1.26	0.65 - 2.41	0.495
Donga	2.05	1.15 - 3.64	0.015	1.96	1.12 - 3.44	0.018
Littoral	1.66	0.83 - 3.32	0.149	1.51	0.73 - 3.14	0.267
Mono	1.49	0.79 - 2.78	0.215	1.58	0.85 - 2.95	0.148
Ouémé	1.31	0.73 - 2.33	0.365	1.24	0.70 - 2.18	0.464
Plateau	2.04	1.10 - 3.79	0.024	2.04	1.13 - 3.70	0.018
Zou	1.11	0.62 - 2.00	0.719	1.21	0.68 - 2.16	0.523

Goodness-of-fit test
F-adjusted test statistic = 0.300
Prob > F = 0.975

Table B3. Factors associated with underweight in children under five, Benin, 2017-2018

**Variables**	**Univariate analysis**			**Multivariate analysis**		
	**cOR**	**95% CI**	**p**	**aOR**	**95% CI**	**p**
**Water**						
No service	1.48	1.25 - 1.77	<0.001	1.15	0.94 - 1.41	0.171
Unimproved	1.05	0.91 - 1.21	0.491	0.89	0.77 - 1.03	0.125
Limited	1.18	0.94 - 1.49	0.157	1.11	0.89 - 1.39	0.362
Basic	1.00			1.00		
**Sanitation**						
No service	1.74	1.42 - 2.14	<0.001	1.14	0.87 - 1.49	0.332
Unimproved	1.50	1.16 - 1.95	0.002	1.12	0.84 - 1.49	0.445
Limited	1.32	1.05 - 1.66	0.016	1.12	0.89 - 1.42	0.329
Basic	1.00			1.00		
**Hygiene**						
No service	1.63	1.27 - 2.09	0.00	1.30	1.00 - 1.70	0.054
Limited	1.65	1.30 - 2.10	0.00	1.33	1.02 - 1.72	0.032
Basic	1.00			1.00		
**Child's age (months)**						
<06	1.00			1.00		
6-11	1.49	1.20 - 1.86	<0.001	1.47	1.18 - 1.84	0.001
12-23	1.63	1.33 - 2.00	<0.001	1.65	1.34 - 2.03	<0.001
24-35	1.45	1.17 - 1.80	0.001	1.53	1.23 - 1.90	<0.001
36-47	1.26	1.01 - 1.57	0.043	1.36	1.09 - 1.71	0.007
48-59	1.36	1.08 - 1.71	0.010	1.48	1.18 - 1.87	0.001
**Child's sex**						
Male	1.14	1.03 - 1.27	0.016	1.14	1.02 - 1.27	0.018
Female	1.00			1.00		
**Child's rank**						
1	1.05	0.91 - 1.21	0.500			
2	1.03	0.89 - 1.19	0.691			
3+	1.00					
**Twin**						
Yes	3.31	2.53 - 4.33	<0.001	3.64	2.75 - 4.81	<0.001
No	1.00			1.00		
**Child's diarrhoea**						
Yes	2.18	1.73 - 2.76	<0.001	1.51	1.28 - 1.79	<0.001
No	1.00			1.00		
**Mother's age**						
15-19	1.28	0.95 - 1.72	0.109	1.39	1.03 - 1.88	0.033
20-29	1.10	0.88 - 1.37	0.397	1.18	0.94 - 1.48	0.148
30-39	0.96	0.77 - 1.21	0.753	1.00	0.79 - 1.25	0.988
40-49	1.00			1.00		

Table B3. continued

Variables	Univariate analysis			Multivariate analysis		
	cOR	95% CI	p	aOR	95% CI	p
**Mother's level of education**						
No education	3.56	1.96 - 6.45	<0.001	2.24	1.20 - 4.19	0.012
Primary	3.22	1.75 - 5.91	<0.001	2.26	1.20 - 4.26	0.011
Secondary	2.43	1.30 - 4.52	0.005	1.85	0.98 - 3.51	0.060
Higher	1.00			1.00		
**Mother's marital status**						
Single	1.21	0.97 - 1.52	0.085			
In couple	1.00					
**Mother's professional activity**						
Yes	1.00					
No	1.09	0.94 - 1.28	0.244			
**Mother's religion**						
Christians	1.00					
Traditional and other	1.21	0.99 - 1.47	0.062			
Islam	1.24	1.06 - 1.44	0.006			
No religion	1.31	0.99 - 1.72	0.058			
**Mother's health insurance**						
Yes	1.00					
No	1.94	0.85 - 4.40	0.114			
**Mother's exposure to newspapers**						
Not at all	1.92	1.19 - 3.11	0.008			
Less than once a week	1.29	0.72 - 2.34	0.394			
At least once a week	1.00					
**Mother's exposure to radio**						
Not at all	1.48	1.29 - 1.70	<0.001	1.35	1.16 - 1.56	<0.001
Less than once a week	1.10	0.92 - 1.30	0.288	1.06	0.89 - 1.26	0.505
At least once a week	1.00			1.00		
**Mother's exposure to television**						
Not at all	1.62	1.38 - 1.90	<0.001			
Less than once a week	1.46	1.21 - 1.77	<0.001			
At least once a week	1.00					
**Household head's sex**						
Male	1.00					
Female	1.03	0.88 - 1.20	0.743			
**Household wealth index**						
Poorest	1.88	1.55 - 2.29	<0.001	1.40	1.05 - 1.87	0.022
Poorer	1.81	1.50 - 2.19	<0.001	1.45	1.10 - 1.92	0.008
Middle	1.46	1.19 - 1.79	<0.001	1.23	0.95 - 1.58	0.115
Richer	1.18	0.96 - 1.44	0.118	1.02	0.81 - 1.29	0.851
Richest	1.00			1.00		

Table B3. continued

Variables	Univariate analysis			Multivariate analysis		
	**cOR**	**95% CI**	**p**	**aOR**	**95% CI**	**p**
**Household size**						
≤5	1.00					
>5	1.10	0.97 - 1.24	0.144			
**Area**						
Rural	1.30	1.12 - 1.51	0.001			
Urban	1.00					
**Department**						
Alibori	2.22	1.58 - 3.11	<0.001	2.27	1.60 - 3.23	<0.001
Atacora	2.11	1.51 - 2.95	<0.001	1.89	1.36 - 2.63	<0.001
Atlantic	1.86	1.35 - 2.57	<0.001	2.37	1.71 - 3.29	<0.001
Borgou	2.18	1.53 - 3.11	<0.001	2.17	1.53 - 3.08	<0.001
Collines	1.00			1.00		
Couffo	1.54	1.08 - 2.20	0.016	1.71	1.20 2.45	0.003
Donga	1.48	1.04 - 2.10	0.029	1.56	1.10 - 2.20	0.012
Littoral	1.34	0.94 - 1.91	0.108	2.27	1.56 - 3.29	<0.001
Mono	1.90	1.34 - 2.72	<0.001	2.06	1.41 - 2.99	<0.001
Ouémé	1.80	1.30 - 2.50	<0.001	2.21	1.58 - 3.10	<0.001
Plateau	2.28	1.59 - 3.27	<0.001	2.40	1.64 - 3.51	<0.001
Zou	1.82	1.32 - 2.50	<0.001	2.13	1.53 - 2.95	<0.001

Goodness-of-fit testtest
F-adjusted test statistic = 1.331
Prob > F = 0.218
